# Harmonized Dual Deep Learning Architectures for Image-Based Diagnostics of Skin Neglected Tropical Diseases: Benchmark Study via Novel Funnel Framework

**DOI:** 10.2196/91544

**Published:** 2026-06-23

**Authors:** Yohannes Minyilu, Mohammed Abebe Yimer, Million Meshesha

**Affiliations:** 1 Faculty of Computing and Software Engineering, Institute of Technology, Arba Minch University, SE, Arba Minch, Ethiopia, 251 0911434681; 2 School of Information Science, College of Natural and Computational Sciences, Addis Ababa University, Addis Ababa, Ethiopia

**Keywords:** skin NTDs classification, 2-stage approach, feature extraction, funnel framework, hyperparameter optimization

## Abstract

**Background:**

While deep learning–based methods are the potential technological solutions for the diagnosis of skin Neglected Tropical Diseases (skin NTDs), limited efforts were seen toward the use of such tools in Ethiopia. Data scarcity, methods, and models selection issues created further challenges in an attempt to close the previous gap.

**Objective:**

This study attempts to design a benchmark image-based diagnostic model for skin NTDs through a synergistic combination of feature extraction pretrained models, a custom-designed convolutional neural network (CNN) model trained on the extracted features, and an integrated data augmentation method applied dynamically.

**Methods:**

For this study, a new skin images dataset is created using skin photographs collected by a team of researchers from the NTDs research center of Arba Minch University Medical College. The new dataset contains 1495 images in 3 classes having severe class imbalance. Extensive experiments were conducted to find the optimal deep learning approach by designing a new CNN model, applying transfer learning, and designing the 2-stage approach that uses pretrained models for feature extraction and trains the new CNN model using the extracted features from the pretrained models and applying data augmentation based on the integrated 2-stage approach. For model selection, the study proposed a novel approach, the funnel framework with cascaded selection of methods and models.

**Results:**

After hyperparameter tuning, the model trained using DenseNet121 feature extractor scored the highest accuracy of 96.6%, *F*_1_-score of 95%, and sensitivity of 95%, while the MNv2-based model scored comparable results of 95.6% accuracy, 90% *F*_1_-score, and 90% sensitivity. This study finally selected the DenseNet121 and MNv2 models for feature extraction to build the final model for skin NTDs classification.

**Conclusions:**

The 2-stage approach significantly boosted the models’ performance compared with other methods, while the data augmentation method further enhanced the performance of the selected models. Finally, this study suggests further studies using advanced class-balancing methods with more data and a possible integration of other clinical data types.

## Introduction

### Background

Neglected Tropical Diseases (NTDs) represent 21 different diseases, including podoconiosis, scabies, and tungiasis, affecting more than 1 billion people globally among underserved communities in the tropical areas [1]. As a tropical country, NTDs are highly prevalent in Ethiopia, with the majority of NTDs identified by the World Health Organization present except for Chagas disease and yaws [2], particularly in the remote areas of the country [3,4]. As estimated by the federal ministry of health [5], more than 75 million people are at risk of contracting at least 1 NTD. Of the 21 NTDs recognized by the World Health Organization, more than half (about 18 of them) have skin manifestations and are called skin NTDs [6-8]. Accordingly, the diagnosis of skin NTDs primarily involves examination of patient skin, presenting opportunities for integrated diagnosis [8], involving the use of artificial intelligence–based skin NTDs diagnostic tools [9]. Although it is not fully explored, previous studies [10-12] have shown that deep learning (DL) methods can be used for the diagnosis of skin NTDs.

This study proposes a DL-based diagnostic model for skin NTDs based on skin images of patients using a new skin images dataset we created for this study. However, apart from the limited previous efforts in DL-based skin NTDs diagnostics, dataset-related issues created challenges in building our proposed model, as the dataset used is characterized by small-sized image samples with a severe class imbalance. Generally, as a computer vision task, the development of a DL-based diagnostic model using skin images requires a large-scale higher-quality skin images dataset [11]. In the case of skin NTDs, data scarcity and dataset-related issues are the major challenges in building DL-based intelligent diagnostic tools for skin NTDs [13,14]. These challenges mainly arise from several factors that include poor record keeping, management, and reporting practices [13]. Additionally, model characteristics regarding usability and efficiency issues are also major challenges in building diagnostic models for skin NTDs. Beyond prediction accuracy, efficiency parameters, such as model complexity, inference speed, response time, and deployment platform options (web-based and mobile-based), are also major factors that determine the selection of DL tools, techniques, and models.

Accordingly, given the data-related issues of the dataset we used for this study and the expected operational platforms, which DL method is appropriate to develop an image-based diagnostic model for skin NTDs? While answering this question requires properly devised strategies based on carefully designed experiments, analysis, and interpretation of results, it establishes a foundational benchmarking effort that helps in identifying suitable DL methods to overcome the mentioned challenges. Therefore, this study conducts extensive experimentations to find the optimal DL solution based on the following guiding questions: (1) Which DL approach (baseline model design, transfer learning, or hybrid) would be a feasible strategy to develop the proposed model? (2) Which DL model architecture would help in developing a high-performance diagnostic model for skin NTDs, given the nature of the dataset used? (3) Which approach helps in creating the robust model development pipeline based on the experimental screening of both DL methods and architectures that collectively address high predictive performance with lower architectural and computational complexity?

While addressing these questions, this study develops a benchmark image-based diagnostic DL model for skin NTDs based on experimentally identified suitable methods and approaches that involve designing a new convolutional neural network (CNN) model and applying transfer learning. Evidently, the data scarcity created higher difficulty in capturing relevant features from input images using baseline models, including the custom-designed model, given the diversity and nature of the manifestations of skin NTDs that include “mossy” limbs in podoconiosis [14]. While the use of transfer learning is ultimately the recommended DL strategy, several factors related to pretrained models create challenges that include huge data requirements, domain incompatibility, tendency of capturing noise features, and lack of a standardized robust diagnostic model development pipeline including skin NTDs. To address such challenges, we designed and implemented the 2-stage approach that presents a robust and extensible architectural pipeline integrating the feature mapping (extraction) models and applying domain adaptation. Furthermore, as the 2-stage approach integrates the 10-layer classification head with different regularization methods, it provides deeper feature filtering architectures.

Overall, this study presents several contributions to the problem domain (skin NTDs diagnostics) and to the field through multiple achievements, which include identification of optimal DL methods for skin NTDs that have higher data scarcity problems; establishment of a robust DL model development pipeline, which incorporates designing the 2-stage approach; methodological rigor that includes robust experimental setup and systematic architectural screening by adopting the funnel framework; and, ultimately, development of DL diagnostic models for skin NTDs, which can serve as an architectural benchmark for skin NTDs (skin-related diseases in general).

### Related Works

Previous studies showed the potential of the DL-based methods for skin NTDs. Accordingly, Steyve et al [10] proposed an optimized real-time diagnostic approach for 3 skin NTDs (Buruli, leishmaniasis, and leprosy) using a support vector machine classifier optimized by a black hole optimization algorithm. Yotsu et al [15] also presented DL methods for using major CNN architectures (ResNet50 and VGG16 models) for the diagnosis of 5 skin NTDs (Buruli ulcer, leprosy, mycetoma, scabies, and yaws). Another study by Pattnayak et al [16] proposed a DL method for 5 skin NTDs (Buruli ulcer, leprosy, mycetoma, scabies, and yaws). Beesetty et al [17] applied a Siamese-based Few Shot Learning model, trained it on an extremely small dataset with fewer disease classes (368 clinically diagnosed leprosy and 28 nonleprosy skin lesions), and reported higher accuracy.

### Challenges Toward Applying DL Methods for Skin NTDs

Multiple factors created challenges toward digitizing the skin NTDs diagnostic procedures using intelligent digital diagnostic tools. Some of the challenges are insufficient infrastructure, data security issues, and limited efforts toward the integration of digital diagnostic tools [18], including the general DL model development challenge, data scarcity, and class imbalance. Multiple large-scale skin image datasets, such as the HAM10000 (Human Against Machine with 10,000 training images) [19] and ISIC (International Skin Imaging Collaboration) [20], are available to train DL models for non-NTD skin diseases. However, it is difficult to find such massive skin image datasets that are publicly available to train DL models for skin NTDs. The other major issues related to data scarcity are the completeness and class distribution imbalance.

### Potential DL Solutions for Intelligent Skin NTDs Diagnosis

Data augmentation, mostly for image classification tasks, is the most widely used machine learning operation to address the problem of data scarcity and distribution imbalance [21-23] through artificially generating images. There are 2 major approaches of data augmentation. The traditional augmentation method uses basic general geometric transformations [21,24], such as cropping, padding, flipping (horizontal or vertical), rotations, permutations, scaling, translations, and addition of noise [23,25]. The cropping, flipping, rotations, and scaling transformations are applied to simulate imaginary variations that might occur in reality. The other data augmentation approach is the class-based conditional augmentation, which is conducted based on predefined conditions either by applying basic geometric transformations or by using generative adversarial (GAN) models [23,26,27].

### Gaps Identified

Overall, the vast literature exploration confirmed that very few efforts were seen toward the use of DL-based diagnostic tools for skin NTDs, specifically in the Ethiopian context. Additionally, the data scarcity issues are the subsequent challenges creating the other major research gaps. These major research gaps clearly suggest that further efforts in the area are mandatory, with a clear indication of having an initially established DL-based diagnostic framework that can serve as a benchmark for current and future studies. Therefore, we conducted this foundational study to develop an image-based skin NTDs diagnostic model using a novel skin NTDs image dataset created by using skin photographs of patients collected from one of the remote and highly affected areas in Ethiopia. We conducted this benchmarking study to identify optimal methods and DL model architectures based on properly designed experimental settings, given the nature of the dataset used for the study. Regarding the dataset-related problems, the study demonstrates the dynamic (online) data augmentation method based on the standard general geometric transformations to initially address the data scarcity problem.

## Methods

### Ethical Considerations

For this study, we created a new dataset using skin photographs of patients with skin NTDs obtained during clinical data collection from a remote affected area in the southwest of Ethiopia. While the data were initially collected by trained health care professionals based on strict ethical procedures using an ethical clearance letter obtained from the institutional review board (IRB) in Arba Minch University (AMU), we acquired the data through institutional research collaboration (“Acknowledgments” section) that warrants full access and use of the firsthand data. To acquire and use the skin images data for this study, proper ethical approval procedures were followed, starting with the acquisition of the ethical clearance letter from the IRB in AMU. Hence, for this study, the authors obtained a specific ethical clearance letter from the concerned institutional review board in AMU (approval protocol number YM23161).

The data collection process was conducted with the full respect of participants’ privacy, where data were acquired from each individual participant based on their will confirmed by a written consent. This was achieved using a dedicated checklist prepared and used to systematically monitor the data collection, ensuring that they were collected based on the free will of each participant. This, specifically, involved a mandatory checklist to ensure that each participant has read and signed the consent form using targeted questions, which include the following: “Does the participant read information statement and willing to sign the consent form?” and the consideration of underaged participants—“if the age is below 18, does the guardian sign the consent form?” Overall, all involved participants provided written consent for their participation and authorized the use of their clinical data for academic purposes, including publications.

As the data collection was performed by a team of trained health care professionals, including public health officers, the entire data collection process was carried out in a professional and responsible manner, authorized by the IRB. Regarding this study, we have been authorized by the same institutional ethical review committee before acquiring and using the data based on a series of verification procedures that include careful analysis of the required data for our study, analysis of participant privacy vulnerability issues as a result of using the data, anonymizing all participant-level data in the images to remove all information in the images that could be used to identify patients (study participants), and strictly complying with ethical requirements for using human patient data—which was confirmed by the ethical clearance letter from the IRB. Therefore, before using the images for this study, all images have been anonymized to remove any personal data to ensure that all participant-identifiable features in any images of the manuscript or supplementary material are not visible.

Finally, as the data were collected from one of the highly affected areas during a mass drug administration (MDA) campaign, the ultimate goal of the data collection was to assess the burden of the skin NTDs that will be used for immediate public health decisions. In this process, the patients living in the affected remote community primarily benefited from the MDA-based data collection, with the patients being diagnosed at the MDA site. However, no special compensations were implemented for the participants as a result of using the data for further study. Additionally, the acquired data are used to build a diagnostic model that intends to serve for diagnosing skin NTDs in the same resource-limited areas.

### Data Collection and Dataset Description

In this study, we used a new handcrafted dataset containing skin photographs of patients with skin NTDs that were captured to show skin areas affected by the skin NTDs. Initially, the data were collected by a team of researchers from the Collaborative Research and Training Center for Neglected Tropical Disease, College of Medicine Health Sciences of AMU. Data were collected in a project-based research for the assessment of skin NTDs burden through community screening during the scabies MDA campaign from Gacho Baba District, Gamo Zone, southwest of Ethiopia. The dataset contains skin photographs (images) of 3 different skin NTDs, namely, podoconiosis, scabies, and tungiasis. These 3 diseases were included in the dataset since they are identified as the most prevalent skin NTDs identified in the specified affected area. The entire data collection process was conducted in a professional and ethical manner, where the whole process was initiated after all legal and ethical issues were addressed, and an ethical clearance was obtained to collect the data. For this study, we acquired the collected data through institutional research collaboration between the NTDs research center of the medical college and computing faculty of the technology institute of AMU. Using the acquired data, we created a new skin NTDs image dataset and used it for this study to develop the proposed DL-based skin NTDs diagnostic model.

### Exploratory Data Analysis

The images were obtained for dermatologist verification and present skin lesions, scratches, excoriations, and other infestations that are typical clinical signs of podoconiosis, scabies, and tungiasis. After acquiring the data, we created 3 separate initial datasets containing the skin images, the unique image IDs, and their corresponding labels for each disease. The final dataset contains 1495 images, as shown in Multimedia Appendix 1. As shown in the statistical distribution, scabies has the largest proportion among the 3 disease classes with a total of 955 instances (955/1495, 63.88%), while tungiasis represents the second largest size with 474 instances (474/1495, 31.71%) and podoconiosis having only 66 instances (66/1495, 4.41%).

### Study Design

**Figure 1.**
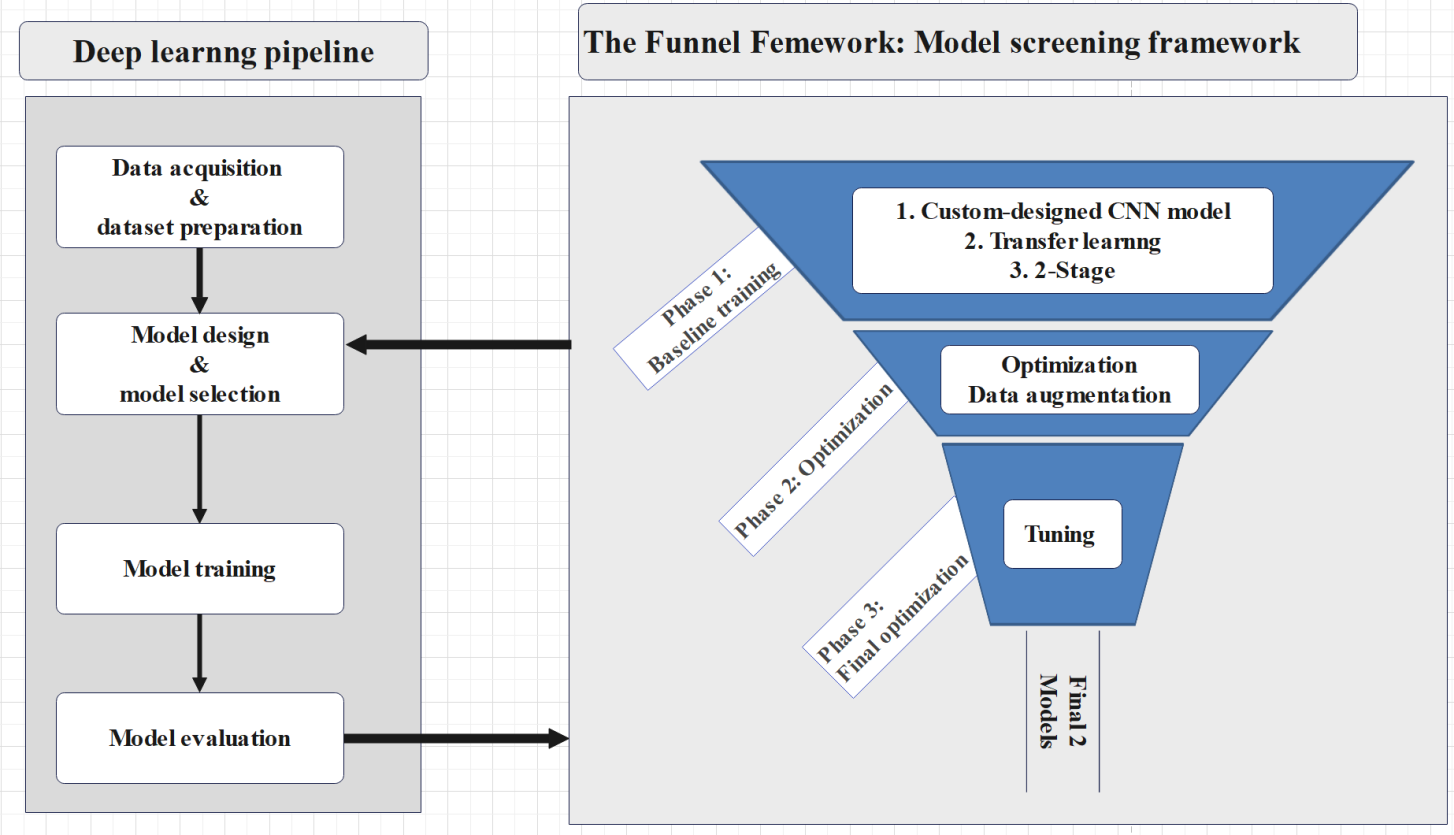
Architecture of the proposed funnel framework based on the cascaded model selection mechanism, CNN: convolutional neural network.

This study intends to conduct a DL architectural benchmarking research that requires a systematic approach to select optimal DL methods and algorithms, and we proposed a mixed research strategy based on the newly proposed funnel framework we adopted for this study, as summarized in Figure 1. Overall, our study needs a systematic approach to select optimal DL methods and models through multistaged experimental filtering. Specifically, the selection of DL pretrained models requires multiple experimentations with systematically devised screening and filtering methods based on comparative analysis of model performance results. Initially, we propose and experiment with 3 different DL methods: first, train a new custom-designed CNN model; second, transfer learning using the 21 selected pretrained DL models; and third, demonstrate the 2-stage approach.

After the completion of these 3 experiments, the best approach and top 5 pretrained DL models will be selected to apply further enhancements to finally select the best 2 models. All these tasks required a systematically designed approach that can be used as a framework to guide the training, screening, and analysis processes. Therefore, we propose a new approach, the funnel framework with cascaded (phased) selection of models and DL methods, as shown in Figure 1. The funnel framework, adapted from the business-related fields, is used to screen out top-performing models and methods initially identified based on a comparative and phased approach. Accordingly, the experiments are conducted in different training settings to select the optimal DL approach that produces comparatively maximum model performance and screen out the high-performing pretrained models in transfer learning and feature extraction.

### Dataset Preparation and Preprocessing

The entire data-splitting process is conducted using the stratified splitting approach with a ratio of 80:20 train-test split, followed by data preprocessing operations. Accordingly, for image resizing, we applied the standard image resolution to all images in the dataset, which required resizing the images to 224 × 224 × 3 pixels for 17 models, while 240 × 240 × 3 pixels and above were used to resize the images for higher EfficientNet models (B1, B3, B5, and V2S). As a next task in data preprocessing, image normalization was programmatically applied to all images.

### Model Development: Model Design and Selection

In this study, we use 3 strategies regarding the development of the proposed DL model, which include (1) training a new custom-designed CNN model, (2) applying transfer learning using selected pretrained DL model architectures, and (3) applying the proposed 2-stage approach—a hybrid approach that uses pretrained DL architectures for feature extraction and training the new CNN model for classification. We applied this set of strategies to experimentally demonstrate possible methods to select the optimal strategy resulting in overall higher performance of models.

### The New CNN Model

**Figure 2.**
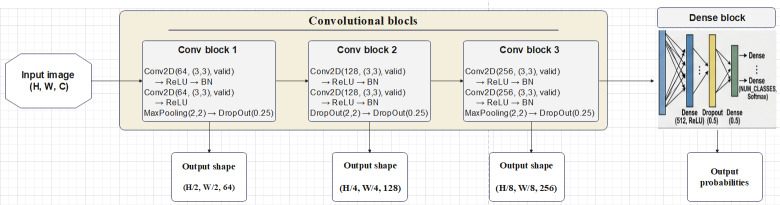
Architecture of the proposed custom-designed convolutional neural network model.

As a first study, we begin our experiments by designing a new custom-designed CNN model that will be used for baseline training and evaluation as well as for the classification of the skin NTDs in the 2-stage approach. Accordingly, we designed a new CNN model consisting of 3 major components (blocks) that represent the 3 different stages of the entire pipeline: feature extraction, the dense layers (including the flattening layer), and the classification head. Based on this general architectural layout, the new model is designed to have 30 layers, containing 8 weight-bearing layers from 6 convolutional and 2 dense layers, 11 regularization layers properly applied across all blocks, 7 activation layers, and 4 spatial refinement layers—3 pooling and 1 flatten layer. Figure 2 visualizes the overall architecture of our custom-designed CNN model.

The model is designed in a consistent manner, implementing a hierarchical structure to capture complex and relevant visual patterns from each input sample image using 3 repeated convolutional blocks having variable filter sizes. Accordingly, the output depth of each block (number of filters) is sequentially doubled across the 3 feature extraction blocks (64 filters in block 1 to 256 filters in block 3). Internally, each feature extraction block is designed to have 2 convolutional layers (Conv2D with 3 × 3 kernels), including properly applied activation, regularization, and dimensionality reduction techniques. Accordingly, the model uses the “relu” activation function, along with robustly implemented regularization methods, which include (1) normalization (BatchNormalization)—applied across all blocks including the dense layers, (2) dropout—applied both in the feature extraction blocks (using Dropout [0.25]) and in the dense block (using Dropout [0.5]), and (3) pooling layers that apply the maximum pooling method—serving dual purposes including regularization, while the maximum pooling method is primarily used for dimensionality reduction [28]. This architectural setup is used to ensure that each feature extraction block extracts and refines features and applies normalization, all before dimensionality reduction (Dropout), to ensure that relevant features and patterns are captured. The overall feature dimension transformations and output shapes of each extraction block are presented in Table 1.

**Table 1.** Architectural summary of the new custom-designed CNN^a^ model.

Model block	Input shape	Convolutional layers	After pooling (2 × 2)	Output
Input layer	224 × 224 × 3	—^b^	—^b^	3
Block 1 (convolutional block 1)	224 × 224	220 × 220	110 × 110	64
Block 2 (convolutional block 2)	110 × 110	106 × 106	53 × 53	128
Block 3 (convolutional block 3)	53 × 53	49 × 49	24 × 24	256
Dense block	Flattened vector	—^b^	512 units	3: number of classes

a CNN: convolutional neural network.

b Not available.

On the final block (classification head), the model applies feature map transformation and final skin NTDs classification using 6 layers: flatten—the layer used to transform the final spatial feature map into a 1D feature vector, fully connected (Dense)—the largest layer in the model having 512 units, regularization—2 independent layers applying BatchNormalization and heavier dropout (Dropout [0.5]), activation layer using “relu,” and output layer—the final layer that predicts the probability distribution among the 3 disease classes (podoconiosis, scabies, and tungiasis) using the SoftMax activation function. Overall, the Adam optimizer and “categorical cross entropy” loss function are used for the final model compilation. All these strategies are properly applied along with a synchronized implementation of early stopping, all to prevent overfitting.

Given the nature of our new dataset, having only 1495 images in the dataset, training a DL model from scratch appeared to be a bit challenging, as the limited size of the feature maps would potentially force the models to learn all the details (including noise pixels) resulting in difficulty to generalize well on new skin NTDs images due to overfitting [28,29]. To overcome this challenge, we conducted further experimental inquiries to identify and use the optimal DL method based on our newly designed CNN model, demonstrating the transfer learning method entirely based on pretrained DL models, followed by the 2-stage approach.

### Transfer Learning

To improve the performance of our baseline CNN model and demonstrate the other potential DL methods, we deployed the transfer learning method by using a diverse set of pretrained DL architectures (CNN and transformer-based). To achieve this goal, we applied a systematic DL architectural selection procedure using a predefined set of model selection parameters to validate, comparatively analyze, and finally select the best model and method for the proposed skin NTDs diagnostic model. Hence, we identified 21 pretrained model architectures, selected based on 4 major selection parameters, which include architectural distribution—defining model family and operational principles (CNN and transformer-based models), model complexity—including both architectural (model size) and computational (efficiency) complexity, and novelty (recency) of models. Table 2 presents a summary of the 21 selected pretrained models.

**Table 2.** Summary of DL^a^ model architectures considered during initial screening.

Model	Architectural family	Core architectural principle(s)	Model complexity		Model efficiency	
			Total parameters	Model size	GFLOPs^b^	Efficiency class
ResNet50	Residual Networks	Skip connections [30]	25.6 M	Large	4.1	Heavy
ResNet18	Residual Networks	Skip connections [30]	11.3 M	Moderate	1.8	Moderate
ConvNext-Small	Modern CNN^c^ Architectures	Transformer-like CNN components using standard ConvNet modules [31]	49.7 M	Large	4.5	Heavy
ConvNext-Tiny	Modern CNN^c^ Architectures	Transformer-like CNN components using standard ConvNet modules [31]	28M	Moderate	4.5	Heavy
CovNeXtv2-Tiny	Modern Pure CNN Architectures	Fully convolutional masked autoencoder framework with a global response normalization layer [32]	28.1M	Large	4.47	Moderate
CovNeXtv2-Atto	Modern Pure CNN Architectures	Fully convolutional masked autoencoder framework with a global response normalization layer [32]	3.7M	Lightweight	0.55	Lightweight
DenseNet121	Densely Connected Networks	Dense CNN (DenseNet) block architecture with feature reuse [33]	7.3M	Moderate	2.9	Moderate
Xception	Depth-wise Separable CNNs	Depth-wise separable convolutions [34]	21.4	Large	8.4	Heavy
EfficientNetB5	Compound-Scaled CNNs	Compound scaling—uniform scale of all dimensions (depth, width, and resolution), built on BMConv^d^ blocks [35]	29M	Large	9.9	Heavy
EfficientNetB3	Compound-Scaled CNNs	Compound scaling—uniform scale of all dimensions (depth, width, and resolution), built on BMConv^d^ blocks [35]	11.2M	Moderate	1.8	Moderate
EfficientNetB1	Compound-Scaled CNNs	Compound scaling—uniform scale of all dimensions (depth, width, and resolution), built on BMConv^d^ blocks [35]	6.9M	Moderate	0.7	Moderate
EfficientNetB0	Compound-Scaled CNNs	Compound scaling—uniform scale of all dimensions (depth, width, and resolution), built on BMConv^d^ blocks [35]	4.4M	Lightweight	0.39	Lightweight
MobileNetV2	Mobile CNNs	Inverted residual blocks with linear bottlenecks [36]	2.6M	Lightweight	0.3	Lightweight
MobileNetV3-Large	Mobile CNNs	Hardware-aware NAS^e^ along with the NetAdapt algorithm (platform-aware adaptation [37,38]	3.2M	Lightweight	0.22	Lightweight
MobileNetV3-Small	Mobile CNNs	Hardware-aware NAS^e^ along with the NetAdapt algorithm (platform-aware adaptation [37,38]	1.1M	Lightweight	0.06	Lightweight
EfficientNetV2B0	Advanced compound-scaled CNNs	Training-aware NAS and scaling—joint optimization of training speed and parameter efficiency [39]	6.2M	Moderate	0.72	Moderate
EfficientNetV2S	Advanced compound-scaled CNNs	Training-aware NAS and scaling—joint optimization of training speed and parameter efficiency [39]	20.7M	Moderate	2.9	Moderate
Recent CNN and transformer-based models						
RepViT	ViT^f^-Inspired Pure lightweight CNN	Reparameterization convolutions in ViT-like Meta-Former structure [40]	5.1M	Moderate	0.80	Lightweight
FasterViT-0	Hybrid (CNN + ViT)	HAT^g^ using window-based self-attention, carrier tokens for local-global representation learning [41]	31.4M	Large	3.34	Moderate
FastViT	Hybrid (CNN + ViT)	RepMixer (structural reparameterization for token mixing) and skip-connection elimination [42]	3.6M	Lightweight	0.70	Lightweight
EfficientViTB0	Hybrid (CNN + ViT)	Lightweight multiscale attention (for context extraction) and MBConv (for local information extraction) [43]	0.7M	Lightweight	0.07	Lightweight

a DL: deep learning.

b GFLOPs: Giga floating point operations.

c CNN: convolutional neural network.

d BMConv: mobile-inverted bottleneck convolution.

e NAS: neural architecture search.

f ViT: vision transformer.

g HAT: hierarchical attention.

All the 21 selected pretrained models were initially trained on ImageNet-1K dataset, initialized with weights from this standard, large, and diversified dataset having around 3.2 million images [44], helping to create general feature extraction baselines. Furthermore, as shown in Table 1, maintaining architectural distribution, we selected the 21 representative models from nine architectural families, which include (1) models of residual networks (ResNet50 and ResNet18) that apply residual learning (or skip connections) method, (2) modern CNN architectures (ConvNextV1 Small/Tiny and ConvNextV2 Tiny/Atto) that have transformer-like CNN components, (3) model with densely connected CNNs (DenseNet121), (4) model with depth-wise separable CNNs (Xception), (5) the EfficientNet family (B0-B5), (6) mobile CNNs MobileNet (V2 and V3), (7) models that apply advanced compound scaling method (EfficientNetV2 B0 and S), (8) recent state-of-the-art lightweight CNN architecture (RepViT), and (9) hybrid transformer-based architectures (FastViT, FasterViT-0, and EfficientViTB0). Regarding architectural and computational complexities, 6 of these models are heavier models having parameters between 30 and 50 million (M) (30 M < parameters < 50 M), while having computational complexities that range between 4.0 and 9.9 Giga floating point operations (GFLOPs). The other 8 models have moderate levels of complexities (6.2 M < parameters < 28.1 M, and 0.7 billion [B] < FLOP < 4.0 B), while 7 of the 21 models are lightweight models (0.7 M < parameters < 5.1 M, and 0.06 B < FLOP < 0.55 B).

### The 2-Stage Approach: Feature Extraction With Integrated CNN Model

**Figure 3.**
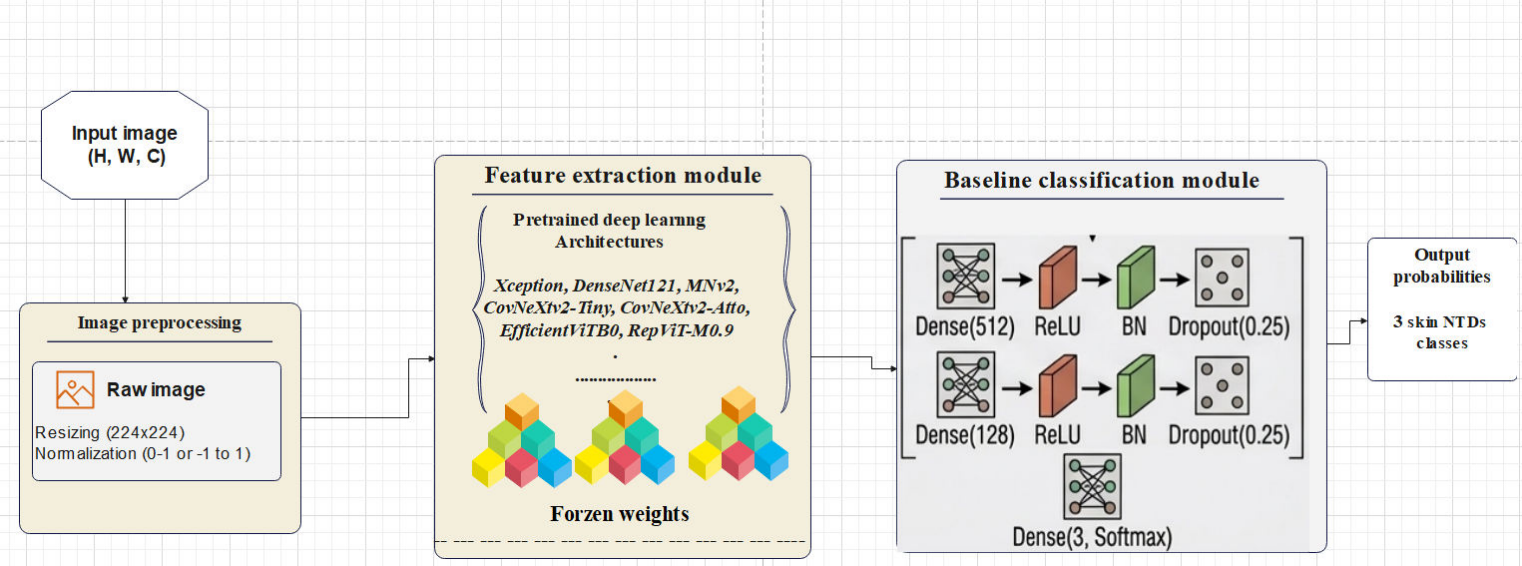
Architecture of the new 2-stage approach. BN: batch normalization; DL: deep learning; skin NTDs: skin Neglected Tropical Diseases.

On the third experimental setting, our proposed 2-staged approach is demonstrated. In this approach, we crafted a robust hybrid model development pipeline that incorporates 2 different DL model architectures, the selected pretrained and our custom-designed CNN models, integrated to the utility modules (data loading, preprocessing, and evaluation). These 2 groups of models are used independently in 2 phases (stages) subsequently operating one after the other to achieve 2 exclusive DL operations, feature extraction (feature mapping) and disease classification (inference), representing the 2 fundamentally isolated but highly interdependent modules in the pipeline. The selected pretrained models are used only for the purpose of feature extraction. Given these 2 modules are highly fundamental that operate subsequently, the disease classification model operates using the output of the feature extraction model, we named the overall hybrid pipeline as the 2-stage approach. Figure 3 presents the overall architecture of our 2-stage approach, depicting the 2 major stages as modules in the DL architecture.

In the first stage (feature extractor module), the selected 21 DL architectures are solely used for feature extraction, which we named them as extractor(s) or feature map(s), and we created 21 specific extractors (maps) by freezing all the trainable weights in each of these 21 models. We use this method to effectively extract features using the mapping logic of each pretrained model and derive feature representations within the high-dimensional feature space (R^2048^) for each input image in our dataset. This method helped us in creating the full feature representations by avoiding pooling layers, as in the case of our custom-designed CNN model, helping us in preserving important spatial representations of features that would potentially be dropped as a result of using pooling method (MaxPooling). After mapping all the input images using the pretrained models, feature matrices are created for each of the separate train and test sets and are prepared to be used by our custom-designed model. This completes the first phase where the feature extraction models completed their only purposes of feature extraction, which are no longer used.

In the second stage (baseline classification module; Figure 3), the feature matrices created using the previously extracted features by each extractor are used to train the new CNN model. However, since our new model was initially designed as a full-fledged CNN architecture, its use in this phase required restructuring. Hence, we restructured our newly designed CNN model by excluding the extraneous feature extraction layers, as these operations are performed by different models in the separate previous module. Guided by our initially designed 30-layer deep and highly regularized CNN model, we specifically redesigned the final 10-layer architecture to enhance the robustness of the classification head, improve classification performance, and add architectural novelty. This approach totally eliminates the 3 feature extraction blocks of the new CNN model, leading to the exclusion of 20 layers. Specifically, this approach directly eliminates the 6 convolutional layers and their corresponding 10 layers (7 activation and 3 maximum pooling), while minimizing the normalization layers to only 3. This creates a final classification model having 10 layers with 3 dense blocks and a final output layer, where each dense block in turn includes regularization methods (normalization and dropout).

Overall, unlike most standard transfer learning pipelines where the pretrained models serve complex tasks with only 1 or 2 classification layers finally added [45], the classification head in our 10-layer customized (redesigned) CNN model has deeper architectural layers. Through its 9 regularization layers, the new CNN head also serves a separate feature filtering purpose, where the final classification layer makes the classification decision using highly relevant skin lesion features.

### Model Training and Evaluation

We used a 5-fold cross-validation method to train and evaluate the models. The metrics used to evaluate the models are selected to assist performance analyses from different perspectives. The macro *F*_1_-score and the class-specific metrics (using sensitivity and specificity) are highly used, as accuracy was found to be a misleading metric due to the highly skewed nature of our new dataset used. Hence, we prioritize the macro *F*_1_-score of models on both the train and test sets to effectively evaluate the models’ generalizability and learning ability. The overall sensitivity (macro recall), the area under the precision-recall curve (AUPRC), and the area under the receiver operating characteristic (AUROC) scores are also highly used to measure how well each model performs in identifying positive disease classes. Furthermore, visual tools using tables, confusion matrices, ROC curves, including performance plots such as the slope charts and radar plots, are also used to analyze models’ performance.

### Final Model Selection

Ultimately, this study selects the final best-performing skin NTDs classification models with the highest classification performance. To achieve this, we applied systematic model selection procedures in 2 phases: first-level selection—applied for the initial feature extraction model screening based on baseline performance score (end of phase 1), and final model selection—applied at the end of model screening experiments (end of phase 2). Overall, the selection of top-performing models involved analysis of performance scores that include macro *F*_1_-score, sensitivity, AUPRC, and 4 class-specific performance metrics (podo-recall, tungiasis-recall, scabies-recall, and podo-*F*_1_-score). Hence, at the end of phase 1, using these extended screening parameters, top-performing models that achieve stable and outperforming scores across the 2 experiments are selected. Accordingly, selected models are used in the next experimental training that applies performance optimization using the dynamic (online) data augmentation method.

Finally, at the end of phase 2, the final 2 best-performing models are selected based on the results achieved during the experiment with the optimization method, and we applied a robust selection procedure based on weighted comparison of performance scores achieved during this last experiment. Accordingly, 6 evaluation metrics (*F*_1_-score [macro], podo-*F*_1_, macro recall [mean], podo-recall, inference speed [samples per second (sps)], and number of parameters) are used, and we applied the weighted scores comparison method with each metric given different weight. To achieve this strategic comparison, we conducted 4 procedures. First, the metrics are categorized as performance (*F*_1_-score [macro], podo-*F*_1_, macro recall [mean], and podo-recall) and efficiency metrics (inference speed and number of parameters). Out of all these metrics, the model complexity (number of parameters) is a metric that is mostly desired to be lower (“lower is good”), while the other 5 metrics represent best model performance when their values are higher (“higher is better”), having opposite directional symmetry with model size. Second, we normalized all values of the 6 comparison metrics using the minimum-maximum normalization method to ensure that all values fall between 0 and 1 and facilitate the combined scores comparison, which includes normalizing the values that already have values between 0 and 1, mainly for (1) avoiding range dilution—minor differences in performance mostly lead to larger ranges that determine comparison; (2) baseline value definition, combined scores mostly perform well with the least score defined as “0.0”; and (3) simplifying directional symmetry. Therefore, to address all these, we applied the minimum-maximum normalization method by defining 2 normalization formulas. First, we normalized the “higher is better” metrics (the metrics that represent best values when their values are higher) using the formula:


$$x_{normalized}=\;\frac{x-x_{\min}}{x_{\max-\;x_{\min}}}$$

where, *x*_normalized_ is a value in any 1 of the 5 metrics (eg, macro *F*_1_-score) representing a single value for a specific model that is being normalized; *x*_min_ is the least value in that specific metric; and *x*_max_ is the maximum score in the same group of metric.

Next, for the “lower is good” metric (model complexity), we applied the normalization using the formula:


$$x_{normalize}=\;\frac{x_{\max-\;x_{\min}}}{x_{\max-\;x_{\min}}}$$

Third, we assigned weights *w* for all evaluation metrics, and as a diagnostic model, disease prediction performance scores are the primary requirements. Hence, we assigned higher weights (0.2) for performance metrics and a relatively lower weight (0.1) for the 2 efficiency metrics (inference speed and number of parameters). Finally, we define a unified objective function that computes the weighted sum of score (WSS) for each model using the formula:


$$\;{WSS}_{m}=\;\sum_{i=1}^{6}w_{i}*x_{i}$$

where WSS_*m*_ is the weighted sum of score of any given model evaluated, *x*_*i*_ is a specific evaluation metric, and *w*_*i*_ is the weight of a given metric. After computing the weighted scores, models are ranked accordingly to identify the top 2 models on the overall performance. Accordingly, the first selected model would be a model with higher complexity, with a consideration of using the model as a back-end (web-based) classification model, while the second selected model would be a lightweight model with the potential of being embedded in edge (mobile) devices for the actual and real-time diagnosis of skin NTDs.

### Experimental Setup

#### Overview

As the study is guided by the funnel framework based on extended experiments, the whole training experiments are conducted in a phased approach having 3 phases. The first phase deals with the establishment of baseline skin NTDs classification models—using the new CNN model, transfer learning, and the 2-stage approach; the second phase applies performance optimization (data augmentation); and the third phase applies the final performance optimization through hyperparameter tuning.

#### Phase 1: Baseline Model Training With Cascaded Model Selection

In this first phase, only baseline skin NTDs classification models are trained in 3 separate experimental setups: the custom-designed CNN model, transfer learning, and the 2-staged approach. This phase is intended for the overall evaluation of baseline models’ performance, which includes comparative analysis and first-level model screening.

##### Custom-Designed CNN Model

The first experimental setting involves training the custom-designed 30-layered CNN model, where the new model is trained and evaluated under 2 different methods. The first training involves the baseline training, where the model is barely trained without applying any advanced machine learning methods for tweaking performance. On the next experiment, the same model is trained by applying data augmentation techniques. These experiments are conducted to evaluate and analyze the performance of this newly crafted model on our new small-sized skin NTDs dataset, as the results from these experiments determine subsequent strategies.

##### Transfer Learning: Baseline Models Using Pretrained Models

On the second experimental setting, we demonstrated the transfer learning method using the selected pretrained DL architectures to build baseline models. Hence, 21 baseline DL models are fully trained on our new skin NTDs image dataset, with proper evaluation of each model on the classification of the skin NTDs, where the results are used for the overall analysis of models’ performance.

##### Two-Stage Approach: Feature Extraction With Integrated CNN Model

On the third experimental setting, the 2-staged approach is demonstrated. To apply this robust training pipeline, we used the 21 systematically selected DL architectures for feature extraction and the restructured new 10-layered CNN model architecture. Hence, 21 different skin NTDs classification models are trained using this approach, where each model is evaluated using the predefined appropriate evaluation methods.

### Phase 2: Performance Optimization

#### Data Augmentation

Evidently, DL models are highly data-intensive and require a large amount of data to achieve excellent performance [46]. Hence, using a relatively small-sized dataset, as the case of this study with only 1495 samples, developing a DL-based diagnostic model with higher classification accuracy is a real challenge; it might not be beneficial at all, compared with the use of large-sized datasets [47]. Methodologically, several DL-based data augmentation methods are available, including the advanced standard and conditional augmentation using GAN models, which are also highly suitable for class imbalance handling. However, their execution requires further experiments, analysis, and selection, including higher computational requirements, which in turn extends this study by deviating from the intended benchmarking objective. Therefore, as a benchmarking study, the dynamic or online (on-the-fly) data augmentation approach [29] is used by applying transformations on the training set to increase the number of training samples by a factor of 5 (including the original training images) and create a total of 4785 training images. This method is primarily used to alleviate the data scarcity issue, analyze performance changes, and increase both the size and the diversity of the training set without the permanent creation of the images. Accordingly, we applied selected geometric transformations such as rotation (0.2), scaling (0.2), and horizontal flipping during model training on the input images at the time of model training to mathematically simulate the real-world diagnostics of skin NTDs. Therefore, using this method, 5 different models are trained based on the 2-stage approach using the final 5 selected extractors.

### Phase 3: The Final Optimization Methods and Hyperparameter Tuning

As the last experimental training, the hyperparameter tuning operation is applied to the final 2 selected models. To achieve this, the “Hyperband” algorithm was used, which is a faster and resource-efficient hyperparameter optimization algorithm than other hyperparameter searching algorithms. Hyperband is an efficient bandit-based Keras algorithm for hyperparameter optimization that uses early stopping with a successive halving algorithm to quickly find good configurations for models [48,49]. Therefore, the hyperparameter tuning operation is applied by running the Hyperband algorithm to the maximum of 30 epochs, with the maximum number of trials being 60. The optimization was carried out based on the validation accuracy, which was set to be the objective metric. Finally, the final best hyperparameters were saved to use for the final optimized training of the skin NTDs classification model.

## Results

This section presents model evaluation results of the 5 experimental settings (baseline training, transfer learning, and the 2-stage approach, including the training with data augmentation and hyperparameter tuning).

### Phase 1: Baseline Model Training With Cascaded Screening

#### The New CNN Model

The new CNN baseline model is trained on the new skin NTDs dataset in 2 different experimental settings, the baseline training that applies no advanced DL method and applying the data augmentation method, as summarized by the overall results in Table 3. Accordingly, on the baseline training, the model achieved an accuracy of 0.674 and *F*_1_-score of 0.42 (with AUROC=0.676 and AUPRC=0.444), with a very high loss (0.978). However, during the second training with data augmentation, the new CNN model achieved improved performance with an *F*_1_-score of 0.446 and an increased loss of 1.458.

However, class-wise, the new model showed the worst sensitivity for podoconiosis (scoring all 0.0 in precision, recall, and *F*_1_-scores), with a macro recall of 0.43 (having a recall of 0.457 and 0.827 for tungiasis and scabies, respectively), as shown in Table 4.

**Table 3.** Overall performance scores of the new CNN^a^ model across the 2 experimental settings.

Experiment	Model/method	Accuracy	Loss	*F*_1_-score (macro)	AUROC^b^ (mean)	AUPRC^c^ (mean)
First	Baseline	0.674	0.978	0.422	0.676	0.444
Second	Dynamic data augmentation	0.691	1.458	0.446	0.790	0.608

a CNN: convolutional neural network.

b AUROC: area under the receiver operating characteristic curve.

c AUPRC: area under the precision-recall curve.

**Table 4.** Class-specific performance of the new CNN^a^ model across the 2 experiments.

Experiment	Model/Method	Recall				*F*_1_-score			
		Podoconiosis	Tungiasis	Scabies	Macro recall	Podoconiosis	Tungiasis	Scabies	Macro *F*_1_-score
First	Baseline	0.0	0.457	0.827	0.428	0.0	0.489	0.776	0.422
Second	Dynamic data augmentation	0.154	0.181	0.979	0.438	0.235	0.296	0.806	0.446

a CNN: convolutional neural network.

With data augmentation, however, the model achieved a macro recall of 0.438 and macro *F*_1_-score of 0.446, with improved class-specific scores in recall (podoconiosis=0.154, tungiasis=0.181, and scabies=0.979) and *F*_1_-score (podoconiosis=0.235, tungiasis=0.296, and scabies=0.806). As confirmed by the results, the use of the standard data augmentation method significantly improved the sensitivity and macro *F*_1_-score of the model for the podoconiosis class (SD +0.109) and (SD +0.017).

#### Transfer Learning: Baseline Performance

On the second and third training settings of the first phase, baseline models are trained using similar pretrained DL model architectures demonstrating (1) the transfer learning method, and (2) the 2-stage approach. Table 5 presents the overall performance of models scored during these 2 experimental settings.

**Table 5.** Performance of the models across the 2 experiments of phase 1 model screening experiments^a^.

Model	Experiment 1: transfer learning (baseline models)					Experiment 2: 2-stage approach (baseline models)				
	Accuracy	Log-loss	*F*_1_-score (macro)	AUPRC^b^ (mean)	Sensitivity (mean)	Accuracy	Log-loss	*F*_1_-score (macro)	AUPRC (mean)	Sensitivity (mean)
ResNet50	0.695	0.733	0.37	0.516	0.39	0.641	1.027	0.26	0.430	0.33
ConvNext-Small	0.641	0.751	0.26	0.552	0.33	0.805	0.532	0.67	0.747	0.62
Xception	*0.973*	*0.106*	*0.94*	*0.975*	*0.91*	*0.94*	*0.219*	*0.9*	*0.937*	*0.88*
EfficientNetB5	0.641	0.785	0.26	0.378	0.33	0.638	0.86	0.34	0.368	0.36
ConvNext-Tiny	0.671	0.71	0.33	0.573	0.37	0.718	0.693	0.43	0.568	0.43
DenseNet121	*0.95*	*0.119*	*0.89*	*0.973*	*0.84*	*0.96*	*0.133*	*0.91*	*0.974*	*0.9*
EfficientNetB3	0.641	0.782	0.26	0.370	0.33	0.668	0.767	0.37	0.429	0.39
MNv3-Large	0.641	0.77	0.26	0.494	0.33	0.681	0.995	0.59	0.625	0.57
MNv3-Small	0.641	0.777	0.26	0.388	0.33	0.708	0.687	0.4	0.640	0.41
EfficientNetB0	0.641	0.786	0.26	0.360	0.33	0.641	1.012	0.26	0.396	0.33
EfficientNetB1	0.641	0.785	0.26	0.363	0.33	0.628	0.828	0.35	0.437	0.37
MNv2	*0.956*	*0.109*	*0.91*	*0.973*	*0.87*	*0.943*	*0.181*	*0.88*	*0.915*	*0.86*
ResNet18	0.641	0.805	0.26	0.352	0.33	0.735	0.737	0.48	0.545	0.49
EfficientNetV2B0	0.641	0.786	0.26	0.349	0.33	0.641	0.793	0.26	0.393	0.33
EfficientNetV2S	0.674	0.694	0.39	0.501	0.4	0.654	0.867	0.44	0.487	0.45
CovNeXtv2-Tiny	*0.95*	*0.124*	*0.919*	*0.983*	*0.886*	*0.94*	*0.222*	*0.876*	*0.935*	*0.862*
CovNeXtv2-Atto	*0.94*	*0.226*	*0.878*	*0.921*	*0.837*	*0.936*	*0.25*	*0.882*	*0.909*	*0.857*
EfficientViTB0	*0.963*	*0.145*	*0.93*	*0.967*	*0.898*	*0.963*	*0.124*	*0.903*	*0.967*	*0.876*
FasterViT0-T8	0.936	0.165	0.883	0.969	0.855	0.671	0.814	0.382	0.469	0.392
FastViT-T8	0.896	0.37	0.602	0.903	0.61	0.93	0.223	0.862	0.939	0.833
RepViT-M0.9	*0.943*	*0.188*	*0.915*	*0.959*	*0.888*	*0.956*	*0.166*	*0.925*	*0.959*	*0.893*

a Values presented in italics represent high-performance

b AUPRC: area under the precision-recall curve.

On the training with only transfer learning (experiment 1, Table 5), only 8 models scored top results, where Xception outperformed all models with the top accuracy (97.3%), macro *F*_1_-score (0.94), and sensitivity (0.91), followed by EfficientViTB0 (accuracy=0.963, *F*_1_-score=0.930, and sensitivity=0.898) and MNv2 (accuracy=95.6%, *F*_1_-score=0.91, and sensitivity=0.87). DenseNet121 and ConvNeXtV2-Tiny are the next high-performing models scoring the same accuracy (95%) and sensitivity (0.84), with ConvNeXtV2-Tiny scoring better *F*_1_-score (0.89). The RepViT, FasterViT, and ConvNeXtV2-Atto models are the other top-performing models with macro *F*_1_-scores of 0.92, 0.88, and 0.84. FastViT, ResNet50, EfficientNetV2S, and ConvNext-Tiny scored macro *F*_1_-scores of >0.33 and average sensitivity >0.37. However, the remaining 9 models similarly scored worst class-specific sensitivity (recall=0.0 for podoconiosis and tungiasis classes), leading to the least macro *F*_1_-score (0.26) and average sensitivity (0.33). Using the 2-stage approach as shown in experiment 2 (Table 5), only 4 models trained using RepViT, DenseNet, EfficientViTB0, and Xception extractors helped their corresponding trained classification models to achieve macro *F*_1_-scores of 0.90 and above, while 8 models scored macro *F*_1_-scores of >086. As shown, the model trained on RepViT-extracted features scored the highest macro *F*_1_-score (0.93, mean sensitivity=0.89), where the model using DenseNet-extracted features had the second highest *F*_1_-score (0.91) with the maximum mean sensitivity (0.90). The model using EfficientViTB0-extracted features scored the next top *F*_1_-score (0.90) during this experiment, followed by the models using the Xception feature extractor (*F*_1_-score=0.90 and sensitivity=0.88), CovNeXtv2-Atto feature extractor (*F*_1_-score=0.88 and sensitivity=0.86), MNv2 feature extractor (*F*_1_-score=88 and sensitivity=0.86), CovNeXtv2-Tiny feature extractor (*F*_1_-score=0.88 and sensitivity=0.86), and FastViT feature extractor (*F*_1_-score=0.86 and sensitivity=0.83).

Method-wise, the use of the 2-stage approach improved the overall performance of the majority of the models, where 62% of the models (13 models) exhibited improved macro *F*_1_-scores, with 2 models showing no variations, while the remaining models exhibited minor declines. Class-wise, the 2-stage approach improved the podo sensitivity (podo-recall) of 5 models (using DenseNet121, MNv3-Large, ConvNeXt-Small, ConvNeXtV2-Atto, and FastViT) by the SD of +0.109, +0.218, +0.218, +0.054, and +0.435, respectively, with 3 models (using ConvNeXtV2-Tiny, EfficientViTB0, and FasterVit) showing declining podo-recall, while the other 13 models showing no changes.

### Best Models Selection

**Figure 4.**
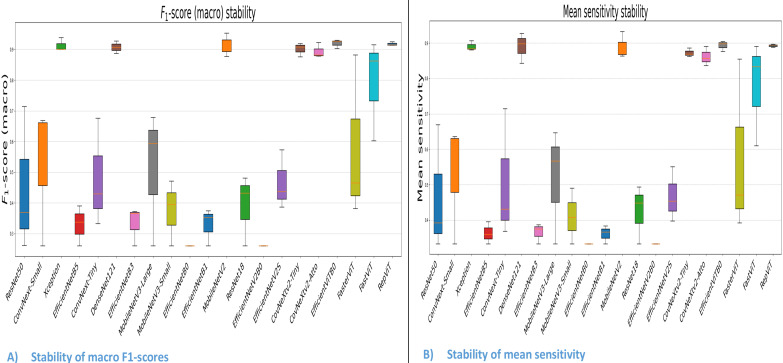
Analysis of models’ performance stability across experiments. (A) Stability of the macro *F*_1_-scores of each of the 21 models across the 2 experiments. (B) Stability of the mean sensitivity scores of the 21 models across the 2 experiments**.**

Overall, the hybrid 2-stage approach applied using the 21 pretrained models (both CNN and transformer-based) yielded superior performance compared with other methods (baseline and transfer learning). Hence, as a DL architectural benchmarking study, we selected this hybrid architecture for further experiments applying optimization methods. However, to screen out short-listed top feature mapping models, we conducted deep and multidimensional analysis that includes performance stability analyses, as shown in Figure 4A and B.

As shown, the box plots clearly depict the stability of the models’ performance primarily in macro *F*_1_-score and overall sensitivity (macro recall) across the 2 experiments. Overall, the models fall into three major categories: (1) stable and least-performing models—models trained using features extracted by EfficientNetB0 and EfficientNetV2B0, where the use of these models for feature extraction resulted in the worst macro *F*_1_-score and sensitivity, while the other 6 feature extractors, EfficientNetB5, EfficientNetB3, MNv3-Small, EfficientNetB1, ResNet18, and EfficientNetV2S, resulted in comparatively better performance scores; (2) the unstable models—models trained on ResNet50, ConvNeXt-Small, ConvNeXt-Tiny, MNv3-Large, FasterViT0, and FastViT-T8, for feature extraction, where the models using these extractors scored highly unpredictable performance scores (macro *F*_1_ and sensitivity), as shown by their wide-range scores (tall boxes); and (3) the stable and top-performing models—representing 7 models trained using the features extracted by Xception, DenseNet, MNv2, ConvNeXtV2-Tiny, ConvNeXtV2-Atto, EfficientViTB0, and RepViT. The models using these 7 pretrained models exhibited minimum variability in their macro *F*_1_-score and sensitivity, while achieving the highest median macro *F*_1_-score (0.88) and median mean sensitivity (0.86) across the 2 experiments, as shown in Figure 4A B.

**Figure 5.**
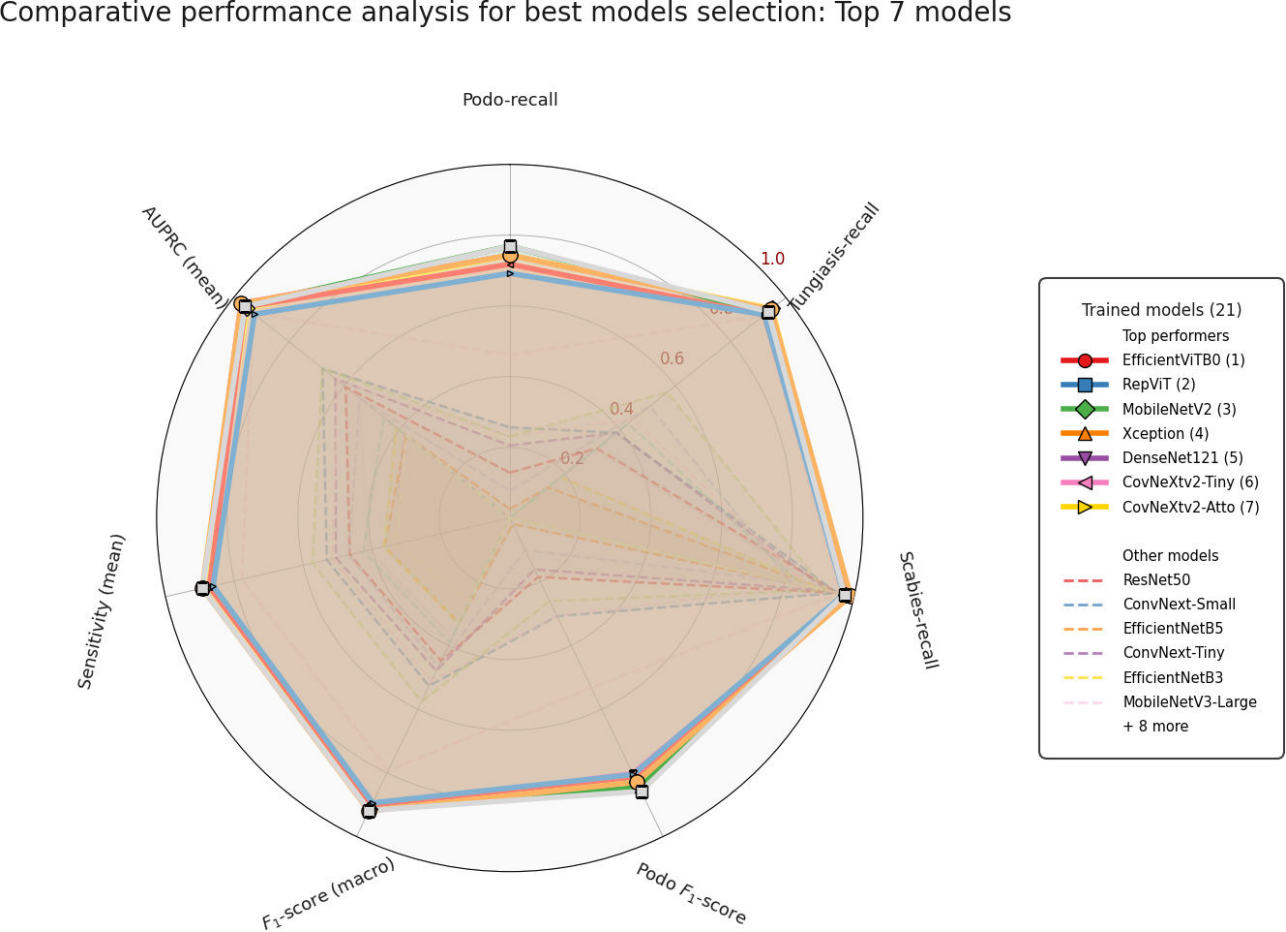
Overall model performance comparison for best model selection. The radar plot illustrates analysis of the models’ performance based on the average scores of the 7 evaluation metrics across the 2 experimental settings, depicting the exceptionally outstanding performance of the 7 top-performing models out of the 21. AUPRC: area under the precision-recall curve.

The next analysis includes performance comparison using 7 parameters, as shown by the radar plot in Figure 5. As shown, only 7 models using EfficientViTB0, RepViT, MNv2, Xception, DenseNet, ConvNeXt-Tiny, and ConvNeXt-Atto feature maps showed exceptionally outperforming performance collectively having a median *F*_1_-score (macro) exceeding 0.95, AUPRC (macro) approaching 1.0, with a comparatively lower median podo-*F*_1_-score approaching 0.90. However, the other 14 models (other than the 7 top-performing models) scored average macro *F*_1_-scores <0.89, average AUPRC<0.92, while showing the worst sensitivity for the minority class with median podo-*F*_1_-score below 0.35.

All these results underlined that the 7 models identified as top-performing models (Figure 5) provided higher feature representation capabilities that led to better overall model performance. Hence, the EfficientViTB0, RepViT, MNv2, Xception, DenseNet, ConvNeXt-Tiny, and ConvNeXt-Atto models are selected for further experiments being used for feature mapping. However, the remaining 14 models were highly challenged to extract useful features that led to poor overall performance and are screened out from being used in further experiments.

### Phase 2: Performance Optimization

On the second phase of our experimental framework, the selected 7 models are retrained by applying data augmentation method, and the results in Table 6 are achieved. In this experiment, the model trained using the MNv2 feature mapping significantly outperformed all other models with macro *F*_1_-score of 0.95 and sensitivity of 0.93, while scoring the fourth highest AUPRC (0.97) and the fourth least errors (loss=0.137). As shown, the models using DenseNet121 and EfficientViTB0 extractors scored the next closer performance, where the model trained using DenseNet121 mapping achieved the second highest macro *F*_1_-score and mean sensitivity of 0.928, while the model using EfficientViTB0 for feature mapping scored a macro *F*_1_-score of 0.926 and mean sensitivity of 0.905.

**Table 6.** Performance of the 7 screened models in the second phase experiment with data augmentation^a^.

Model	Accuracy	Log-loss	*F*_1_-score (macro)	AUPRC^b^ (mean)	Sensitivity (mean)
Xception	0.94	0.158	0.901	0.982	0.884
DenseNet121	0.966	0.13	0.928	0.974	0.928
MNv2	*0.973*	*0.137*	*0.953*	*0.967*	*0.933*
CovNeXtv2-Tiny	0.956	0.128	0.908	0.956	0.869
CovNeXtv2-Atto	0.953	0.209	0.922	0.944	0.891
EfficientViTB0	0.97	0.098	0.926	0.985	0.905
RepViT-M0.9	0.956	0.168	0.914	0.959	0.897

a Values presented in italics represent high-performance models.

b AUPRC: area under the precision-recall curve.

Overall, during this experiment, the 7 feature extraction pretrained models (MNv2, DenseNet121, EfficientViTB0, CovNeXtv2-Atto, RepViT, CovNeXtv2-Tiny, and Xception) helped their corresponding skin NTDs classification models to score macro *F*_1_-scores >0.90, AUPRC >0.94, and mean sensitivity >0.86, with overall loss ≤0.21.

### Final Models Selection

For the systematic selection of the final 2 best feature extraction pretrained models that provide the best feature mapping for the skin NTDs classification models, we applied the robust weighted score comparison method defined in the “Methods” section of the study. Hence, applying our predefined WSS formula, we computed the WSS for the scores achieved on the third experiment that applies data augmentation and for the overall average performance of the models across the 3 experiments. For each group of evaluation, we applied two different sets of weights for the metrics: (1) weights given only to the predictive performance metrics—equal weights of 0.25 are given to only the 4 predictive metrics (macro *F*_1_, recall, podo-recall, and podo-*F*_1_), while the speed and number of parameter are not considered to evaluate only predictive performance; and (2) weights given to all the 6 metrics—the predefined weights of 0.2 given for the 4 predictive performance metrics, while the efficiency metrics are given the weights of 0.1. Table 7 summarizes the overall results of the combined weighted evaluation method.

**Table 7.** Combined weighted method results for the 7 prescreened models (final model selection)^a^.

Model	Performance metrics				Efficiency metrics		Weighted scores (optimization)		Weighted scores (average)	
	*F*_1_-score (macro)	Podo-F1	Macro recall (mean)	Podo-recall	Speed (sps)	Parameters	WSS^b^ (predictive)	WSS (overall)	WSS (predictive)	WSS (overall)
RepViT-M0.9	0.05	0.03	0.09	0.10	0.09	0.10	0.335	0.459	*0.974*	*0.975*
DenseNet121	*0.10*	*0.06*	*0.18*	*0.20*	*0.07*	*0.06*	*0.681*	*0.671*	0.511	0.528
EfficientViTB0	0.10	0.03	0.11	0.10	0.00	0.06	0.424	0.399	*0.784*	*0.772*
Xception	0.00	0.03	0.05	0.10	0.03	0.00	0.222	0.211	0.855	0.684
CovNeXtv2-Atto	0.08	0.10	0.07	0.10	0.10	0.10	0.442	0.554	0.009	0.204
MNv2	*0.20*	*0.20*	*0.20*	*0.20*	*0.07*	*0.04*	*1.0*	*0.916*	0.688	0.685
CovNeXtv2-Tiny	0.03	0.00	0.00	0.00	0.05	0.07	0.034	0.155	0.241	0.367

a Values presented in italics represent high-performance models.

b WSS: weighted sum of scores.

As shown, the model using the MNv2 feature mapping showed an exceptionally highest combined weighted score with an overall WSS of 0.92 on the aggregate weighted scores. This model still showed an exceptionally outperforming combined weighted score on the predictive performance metrics with a WSS of 1.0. The other model using the DenseNet121 mapping has the second highest weighted scores having an overall WSS of 0.67 and a WSS of 0.68 on the predictive performance. On the same experiment, the models using CovNeXtv2-Atto (0.55) showed the next top overall WSS, followed by the model using RepViT-M0.9 extractors (0.46). On this last experiment, the hybrid transformer-based architecture EffcientViTB0 showed lower weighted scores (0.399 and 0.424) on the overall and predictive WSS. Conversely, using the aggregated performance derived by computing the average scores of each metric for all models across 3 experiments, the combined weighted scores present a completely different set of scores. Based on these results, the model using the RepViT-M0.9 extractors showed an exceptionally higher combined weighted scores both on the overall WSS (0.98) and the predictive WSS (0.97), followed by the model using EfficientViTB0 with an overall WSS (0.98) and predictive WSS (0.97). The models based on MNv2 and DenseNet121 showed the next higher weighted scores (overall WSS of 0.69 and 0.53, respectively) on the aggregated scores of the models across the 3 experiments.

Overall, the data augmentation method is applied to optimize the models’ performance, given the study faced the data scarcity problem. However, the models using the RepViT-M0.9 and EfficientViTB0 extractors unexpectedly reacted negatively to this optimization method that expands the sample size and data variance, while the same method significantly boosted the performance of the models based on the MNv2 and DenseNet121 feature mapping. These results reveal a generalization paradox problem, showing (1) overspecialization—the models based on the RepViT-M0.9 and EfficientViTB0 extractors are experiencing performance instability and a potential overfitting due to memorization of the initial small-sized training data, and (2) latent generalization—the models using the MNv2 and DenseNet121 extractors showed their hidden generalization abilities that are exposed as the result of the data augmentation method. Therefore, based on these major driving facts, we selected the MNv2 and DenseNet121 pretrained models for feature mapping in our final skin NTDs diagnostic model.

### Phase 3: The Final Optimization

On the final experiment, hyperparameter tuning is applied on the last 2 models selected, which helped the model using the DenseNet121 feature extractor achieve improved performance, as shown in Table 8.

**Table 8.** Performance scores of the final 2 selected models after hyperparameter tuning^a^.

Model	Accuracy	Macro *F*_1_-score	Loss	AUROC^b^ (average)	AUPRC^c^ (average)	Recall				*F*_1_-score			
						Podoconiosis	Tungiasis	Scabies	Macro recall	Podoconiosis	Tungiasis	Scabies	Macro *F*_1_-score
DenseNet121	*0.966*	*0.946*	*0.181*	*0.996*	*0.974*	*0.923*	*0.979*	*0.974*	*0.959*	*0.889*	*0.968*	*0.982*	*0.946*
MNv2	0.96	0.906	0.150	0.987	0.955	0.769	0.947	0.979	0.898	0.80	0.937	0.982	0.906

a Values presented in italics represent high-performance models.

b AUROC: area under the receiver operating characteristic curve.

c AUPRC: area under the precision-recall curve.

**Figure 6.**
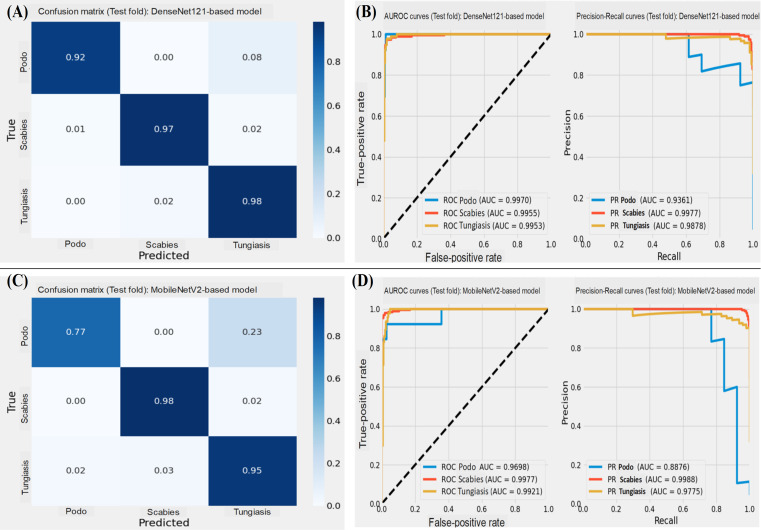
Comprehensive classification evaluation of the final 2 models. (A) Confusion matrix showing class-wise results for the model trained using DenseNet121 extractor, (B) AUROC and area under the precision-recall curve (AUPRC) of the model using DenseNet121 feature extractor, (C) confusion matrix showing class-wise results for the model trained using MNv2 extractor, and (D) AUROC and AUPRC of the model using MNv2 feature map. AUC: area under the curve; AUROC: area under the receiver operating characteristic curve; PR: precision-recall; ROC: receiver operating characteristic.

Figures 6A-6D summarizes the overall performance of the final 2 models trained using the DenseNet121 and MNv2 feature extractors after hyperparameter tuning.

After hyperparameter tuning, the DenseNet121-based model scored improved performance, compared with the previous experiment with data augmentation, achieving an accuracy of 0.966, *F*_1_-score of 0.95 (Δ=+0.018), and mean sensitivity of 0.96 (Δ=+0.031). However, the MNv2-based model exhibited declining performance in *F*_1_-score (Δ=−0.047) and macro recall (Δ=−0.035), including podo-specific recall and *F*_1_-score.

## Discussion

### Principal Findings

As a DL architectural benchmarking effort, this study developed a diagnostic model for skin NTDs based on the funnel framework with an extensive experiment-based cascaded architectural screening. A significant portion of the experimental design involved identifying robust pretrained DL architectures for feature mapping to be used with our custom-designed CNN model. On average, across the first 2 phases (5 experiments), the custom-designed CNN model and 10 other models trained using the feature mapping architectures yielded poor overall performance with macro *F*_1_-scores below 0.50, 5 models (using MNv3-Large, ConvNext-Small, FasterViT, FastViT, and CovNeXtv2-Atto extractors) showed moderate performance with above 0.50, and 6 models using EfficientViTB0, RepViT, MNv2, Xception, DenseNet121, and ConvNeXtV2-Tiny extractors achieved exceptionally high performance with *F*_1_-scores exceeding 0.90 and sensitivity above 0.87. This exceptionally high performance of the top 6 models is attributed to their robust feature mapping logic that allowed deriving high-dimensional representative features, given our small-sized skin NTDs dataset. Method-wise, the 2-stage approach applying data augmentation resulted in performance improvements for the majority of the models, which underscore the success of the 2-stage approach for skin NTDs classification, given the dataset problems.

The other critical finding of this study highlighted that modern and state-of-the-art DL architectures such as EfficinetNetB5 and EfficinetNetB3, even the highly anticipated lightweight models of EfficinetNetB0 variants, MNv3-Large and MNv3-Small, were unable to extract useful features from the dataset used. All these models proved to be highly data-intensive and extremely sensitive to class imbalance. Conversely, DenseNet121, MNv2, Xception, including the comparatively recent DL architectures of ConvNeXtV2 (Tiny/Atto), and RepViT provided highly robust feature mapping capability, while the transformer-based hybrid architecture EfficientViTB0 showed its potential. Ultimately, the DenseNet121 and MNv2 demonstrated their robustness across the 5 different experimental trainings, including the final optimization. Hence, these 2 models are selected due to their overall feature mapping capability to be integrated with our custom-designed CNN model. Overall, the success of the DenseNet121 model is architecturally linked to its dense connectivity patterns and inductive biases. These factors allowed the trained classifier to reuse feature maps from previous layers through dense blocks, which expands the feature maps into more representative data. Likewise, the success of the MNv2 models is attributed to its internal information bottleneck method. To combat the dataset-related problems such as high variance and possible data memorization, several regularization strategies were synergistically applied. The strategies included data augmentation, batch normalization, dropout, and early stopping with a synchronized implementation of callbacks. While these methods successfully improved model generalization on unseen test sets, regularization alone was insufficient to fully address the overfitting issues caused by the severely skewed distribution of disease classes. Specifically, for the 12 models excluded after the first phase experiments, all these regularization methods were unable to improve performance even with the application of data augmentation. This clearly proves that the severe class imbalance, especially between scabies and podoconiosis, greatly affected the models’ performance. This clearly requires experimental investigation by applying the different methods for class balancing, both at the data and algorithm levels.

### The Synergistic Hybrid Approach

Overall, the most effective strategy identified was the 2-stage hybrid approach that combined the high-performing feature extractors with the properly designed CNN classification module. This final harmonized integration involved 3 key strategies: the robust feature extraction architectures, the optimal CNN classification head configuration, and the dynamic data augmentation methods. The harmonized use of all these 3 strategies, along with hyperparameter tuning, provided high-performance classification models by ensuring that each model operated on the optimized output of its preceding module. Ultimately, this study selected the final 2 skin NTD diagnosis models that achieved an *F*_1_-score > 0.95 using MNv2, and an *F*_1_-score > 0.93 using DenseNet121 after data augmentation. All these higher-performance scores are attributed to the combined use of all DL methods, and feature mapping models we use based on our 30-layered very deep CNN model have brought tangible disease-predictive performance improvements across all models.

The integrated 2-stage approach demonstrated its potential for skin NTDs diagnosis, given the study used a dataset with severe class imbalance and small sample images. While most of the DL methods and pretrained models used in the 2-stage approach are preexisting, the harmonization of the feature mapping with our 10-layered classification head created a different DL architecture. This architecture allowed us to establish information bottlenecks using the compact, highly focused, and properly regularized 10-layer classification head that trains on the extracted features. This new architecture added robustness to our model development pipeline, allowing us to build top-performance skin NTDs diagnostic models using the highly constrained dataset, compared with the standard transfer learning method using the same pretrained models. Accordingly, the improved sensitivity of models for the minority class (podo-recall) confirmed the achievements of the architecture, where 5 models showed their sensitivity for podoconiosis, while 62% of the models (13 models) have shown lower overall performance and no variation in podo-recall. These facts clearly underline that the 2-stage approach provided methodological solutions to the severe class imbalance problem. Furthermore, the approach also demonstrated domain adaptation through its two separate modules: (1) feature extraction module—the feature mapping logics of the models initially trained on ImageNet-1K adapted for our skin NTDs diagnostic model, where these diseases have diverse lesion types and textures such as “mossy limbs” that require symmetric analysis of limbs to detect podo based on swellings; and (2) feature refinement module—the highly regularized 10-layered classification head that applies further refinements on the feature matrices extracted by the mapping models.

### Clinical Relevance and Further Considerations

This study developed benchmark image-based diagnostic model(s) for skin NTDs using the traditional augmentation method to initially alleviate data scarcity issues (limited sample images with severe class imbalance). We applied this method on our sample input image using our predefined basic geometric transformations at the time of model training to mathematically simulate the real-world diagnostics of skin NTDs. In the actual real-world scenarios, skin NTDs are highly prevalent in remote underserved areas, where the diagnostics of the diseases are mostly undertaken by middle-level health care workers under low-resource settings. Hence, our model design assumed these scenarios that our model is used by middle-level health care workers without the need for highly sophisticated devices, where moderate-quality handheld smartphones can properly serve the diagnostics. Accordingly, we added the basic transformation techniques to our augmentation pipeline that includes rotation (20 degrees), shearing (range=0.1), and zooming (range=0.1) to avoid variations in our models’ predictions that can be caused by variable camera angles, inconsistent framing, and varying focal distances in photographs of the skin NTDs. We also added the horizontal flipping transformation to maintain anatomical symmetry, and random brightness adjustments are also applied to simulate the diagnostic practices that can be conducted under different lighting adjustments.

While the study achieved its objective, the data scarcity issues still remained to be the major challenges. However, the top performance scores achieved by the final 2 optimized models (the models using the MN2 and DenseNet121 feature maps) underlined that further experiments using advanced DL-based data augmentations and balancing methods have the potential of further improving the predictive performance of the models. Hence, using each of the selected benchmark models as a basis, advanced DL-based methods such as the use of GAN-generated samples and weighted balancing methods are expected to significantly boost the models’ performance, as confirmed by the performance improvements achieved by applying the traditional dynamic data augmentation.

Additionally, as a clinical diagnostic model, the other important aspect expected from such systems is the treatment facility, once the diseases are correctly identified with higher diagnostic accuracy. Generally, the incorporation of treatment recommendations represents the deployment-ready (final) stage of a diagnostic tool, mostly indicating that the model passed several improvement and validation stages. It also requires further system validation and authorization. Therefore, marking the current gaps, incorporation of treatment recommendations, and performance improvements using advanced DL methods are the immediate research tasks.

### Conclusions

Ultimately, this study developed a benchmark image-based diagnostic model for skin NTDs through a robust hybrid DL pipeline using a novel skin NTDs dataset collected from a remote area representing an underserved community in the Southwest of Ethiopia. The study developed a new baseline model, applied transfer learning, designed a 2-stage approach, and applied dynamic data augmentation, where all experiments were conducted based on a novel research framework proposed for this study, the funnel framework. Using the funnel framework, optimal methods and high-performing models were selected in a phased approach. With the 2-stage approach being the best model-building approach, the DenseNet121 and MobileNetV2 were top-performing feature extractors. Finally, after the last training applying hyperparameter tuning, the models trained using these extractors still showed performance improvements, DenseNet121 (accuracy=96.6% and *F*_1_-score=0.85) and MobileNetV2 (accuracy=96.6% and *F*_1_-score=0.85). Therefore, the study ultimately selected the DenseNet121 and MobileNetV2 models for feature mapping (extraction) for the final DL-based skin NTDs diagnostic models.

While we developed the intended model for the diagnosis of skin NTDs using the novel skin images dataset, this study also exhibits some downsides that limit the study from contributing to its potential. Data scarcity and severe class imbalance are the primary challenges. The number of diseases represented in this study is also limited to only 3 diseases while there are more than 8 skin NTDs prevalent in Ethiopia. Additionally, the study does not demonstrate data-balancing methods due to resource-related constraints and the extended experiments required. Furthermore, only image data were considered to develop the proposed diagnostic model. Therefore, we recommend further efforts to address the mentioned limitations. First, it is recommended that additional data must be collected from different affected areas (if applicable) with the data being representative of all disease classes. Additionally, DL-based class-balancing methods, such as conditional augmentation based on generative models, are recommended to be experimented. We also suggest the inclusion of multiple types of data, such as text-based patients’ data, to expand the dimensionality of the dataset used.

## Supplementary material

10.2196/91544 Multimedia Appendix 1 Distribution of data samples in the new skin Neglected Tropical Diseases image dataset. The figure illustrates data distribution portraying class imbalance among the 3 disease classes: scabies (dark blue, 63.9%), tungiasis (teal, 31.7%), and podo (light green, 4.4%).

## Abbreviations

AMU: Arba Minch University
AUPRC: area under the precision-recall curve
AUROC: area under the receiver operating characteristic curve
CNN: convolutional neural network
DL: deep learning
GAN: generative adversarial
GFLOP: Giga floating point operation
IRB: institutional review board
ISIC: International Skin Imaging Collaboration
MDA: mass drug administration
NTD: Neglected Tropical Disease
skin NTD: skin Neglected Tropical Disease
WSS: weighted sum of scores
