# Supplementary figures and images for: Harmonized Dual Deep Learning Architectures for Image-Based Diagnostics of Skin Neglected Tropical Diseases: Benchmark Study via Novel Funnel Framework
Source: JMIR Dermatol. 2026 Jun 23;9:e91544. doi: 10.2196/91544 (PMC13288414; doi:10.2196/91544)

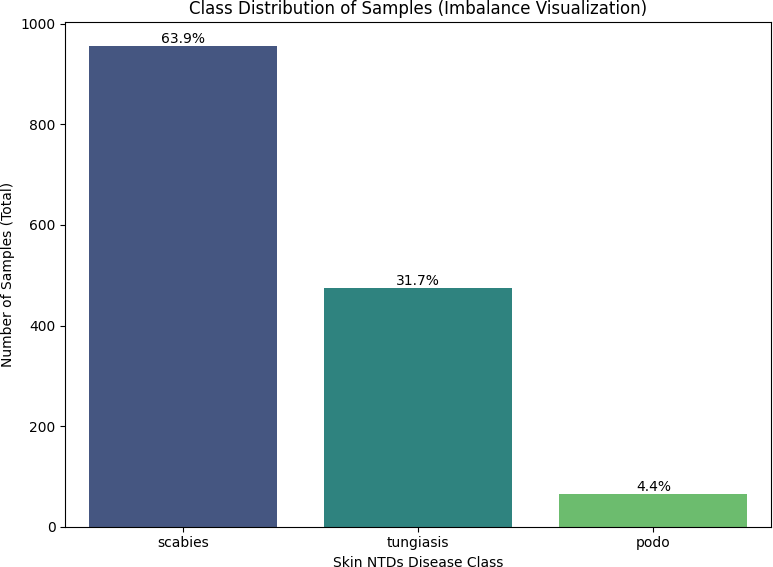

Supplement: Multimedia Appendix 1 [file derma-v9-e91544-s001.png]
